# Crystal structure of NiFe(CO)_5_[tris(pyridyl­meth­yl)aza­phosphatrane]: a synthetic mimic of the NiFe hydrogenase active site incorporating a pendant pyridine base

**DOI:** 10.1107/S2056989019003256

**Published:** 2019-03-11

**Authors:** Natwara Sutthirat, Joseph W. Ziller, Jenny Y. Yang, Zachary Thammavongsy

**Affiliations:** a Department of Chemistry, University of California, Irvine, Natural Sciences II, Irvine, CA 92697, USA

**Keywords:** crystal structure, NiFe hydrogenase, biomimic, TPAP, hydrogen bonding, offset π–π inter­actions, C—H⋯π inter­actions, supra­molecular framework

## Abstract

The reaction of Ni(TPAP)(COD) {where TPAP = [(NC_5_H_4_)CH_2_]_3_P(NC_2_H_4_)_3_N} with Fe(CO)_5_ resulted in the isolation of the title heterobimetallic NiFe(TPAP)(CO)_5_ complex di-μ-carbonyl-tricarbon­yl[2,8,9-tris­(pyridin-2-yl­meth­yl)-2,5,8,9-tetra­aza-1-phosphabi­cyclo­[3.3.3]undeca­ne]ironnickel, [FeNi(C_24_H_30_N_7_P)(CO)_5_]. Characterization of the complex by ^1^H and ^31^P NMR as well as IR spectroscopy are presented. The structure of NiFe(TPAP)(CO)_5_ reveals three terminally bound CO mol­ecules on Fe^0^, two bridging CO mol­ecules between Ni^0^ and Fe^0^, and TPAP coordinated to Ni^0^. The Ni—Fe bond length is 2.4828 (4) Å, similar to that of the reduced form of the active site of NiFe hydrogenase (∼2.5 Å). Additionally, a proximal pendant base from one of the non-coordinating pyridine groups of TPAP is also present. Although involvement of a pendant base has been cited in the mechanism of NiFe hydrogenase, this moiety has yet to be incorporated in a structurally characterized synthetic mimic with key structural motifs (terminally bound CO or CN ligands on Fe). Thus, the title complex NiFe(TPAP)(CO)_5_ is an unique synthetic model for NiFe hydrogenase. In the crystal, the complex mol­ecules are linked by C—H⋯O hydrogen bonds, forming undulating layers parallel to (100). Within the layers, there are offset π–π [inter­centroid distance = 3.2739 (5) Å] and C—H⋯π inter­actions present. The layers are linked by further C—H⋯π inter­actions, forming a supra­molecular framework.

## Abstract 2

The synthesis and the crystal structure of a NiFe complex containing an aza­phosphatrane ligand is discussed. The NiFe(TPAP)(CO)_5_ complex contains a pendant pyridine base in close proximity to the NiFe metal center.

## Chemical context   

Rare and expensive metals such as Pt are often used to catalyze the production and oxidation (for utilization in fuel cells) of H_2_. Because of this, the production and utilization of H_2_ for clean energy applications has motivated scientists to produce efficient and cheap H_2_ evolution catalysts. In nature, hydrogenase enzymes catalyze the reversible production and oxidation of H_2_ with the metals, Ni and Fe (Lacasse & Zamble, 2016 ▸). Inspired by nature, this work aimed to structurally mimic the active site of the NiFe hydrogenase enzyme (Kaur-Ghumaan & Stein, 2014 ▸). NiFe hydrogenase contains an NiFe active center, where Fe is coordinated with three different types of ligand (C≡O, C≡N, and a sulfur atom) while Ni is coordinated by four cysteine residues. The C≡O, C≡N and the sulfur-atom ligands play a role in maintaining the oxidation state of Fe^II^ and stabilizing the oxidation state changes of the Ni ion during the catalytic cycle (Behnke & Shafaat, 2016 ▸). In our previous work, the transannular inter­action of bridgehead N and P atoms in the tri(pyridyl­meth­yl)aza­phosphatrane (TPAP) ligand was investigated for the stabilization of metal ions in different oxidation states (Thammavongsy *et al.*, 2018 ▸). A recent study by Johnson and co-workers found that the transannular inter­action in aza­phosphatranes plays a potential role in Pd cross-coupling reactions, where the oxidative addition event ‘is promoted due to electron donation to the metal center from transannulation’ (Matthews *et al.*, 2018 ▸). The transannular inter­action in TPAP could play a similar role in stabilizing the Ni ion. Additionally, a study by Armstrong and collaborators found a conserved arginine residue was vital for catalysis in NiFe hydrogenase (Evans *et al.*, 2016 ▸). They propose the guanidine base of arginine participates in activation of H_2_. As a result of this conserved motif, incorporation of pendant bases into the ligand design of synthetic models of NiFe hydrogenase is important, but has been rarely observed in reported synthetic models of NiFe hydrogenase (as opposed to those of FeFe hydrogenase). In the title complex, NiFe(TPAP)(CO)_5_, whose synthesis is illustrated in the reaction scheme below, the TPAP ligand features a pendant pyridine base, providing a close structural mimic of the NiFe hydrogenase enzyme.
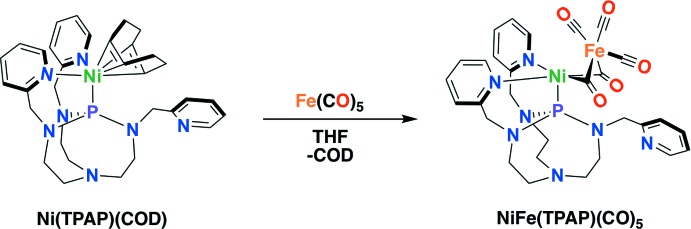


## Structural commentary   

**Figure 1:**
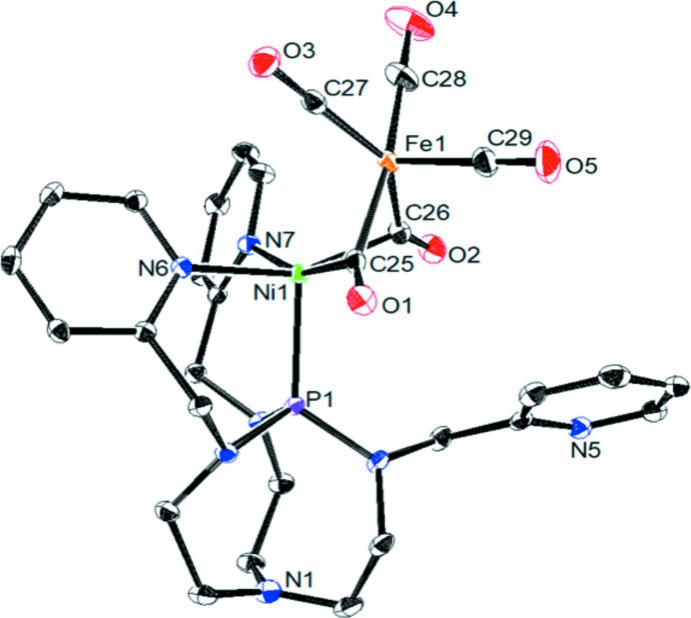
The mol­ecular structure of complex NiFe(TPAP)(CO)_5_, with atom labelling. The displacement ellipsoids are drawn at the 50% probability level. For clarity, the hydrogen atoms have been omitted. **[All the atoms must be labelled]**

The title heterobimetallic NiFe(TPAP)(CO)_5_ complex (Fig. 1 ▸), displays two bridging CO mol­ecules between the Ni and Fe metal centers. Selected bond lengths and bond angles are given in Table 1 ▸. The Fe^0^ center shows a five-coordinate pseudo square-pyramidal geometry comprising three terminally bound CO and two bridging CO mol­ecules. The τ_5_ value of the Fe^0^ atom is 0.40, where τ = 0 represents an ideal square pyramidal and 1 represents an ideal trigonal–bipyramidal geometry (Addison *et al.*, 1984 ▸). The Ni^0^ center is also coord­inated by the two bridging CO mol­ecules and the TPAP ligand, where the two nitro­gens from two pyridines and the phosphorus of the aza­phosphatrane are coordinated. The Ni^0^ ion displays a five-coordinated square-pyramidal geometry with a τ_5_ value of 0.06. The bond lengths of the CO mol­ecules bridging between the Ni and Fe ions are 1.1821 (16) and 1.1754 (17) Å for O1—C25 and O2—C26, respectively. These bond lengths are longer than the terminally bound CO mol­ecules on Fe, which are 1.1509 (17), 1.148 (2) and 1.1531 (19) Å for O3—C27, O4—C28 and O5—C29, respectively. The shorter bond distances in the bridging CO mol­ecules is indicative of π-back-bonding from the two metal centers to the bridging CO ligands. The Ni—Fe bond length is 2.4828 (4) Å, similar to the Ni—Fe bond length (∼2.5 Å) in the reduced state of NiFe hydrogenase (Garcin *et al.*, 1999 ▸). The distance between atoms P1 and N1 in TPAP is 3.2518 (13) Å, consistent with a fully relaxed, pro-form of aza­phosphatrane (Verkade, 1993 ▸). One pyridine group from TPAP is uncoordinated to the Ni or Fe metals. Atom N5 of the non-coordinating pyridine is not facing directly towards the metal ions, resulting in an approximate distance of 5.61 and 5.93 Å from Ni and Fe, respectively. In comparison, the argin­ine side chain lies less than ∼4.5 Å from both the Ni and Fe in NiFe hydrogenase (Evans *et al.*, 2016 ▸).

## Supra­molecular features   

**Figure 2:**
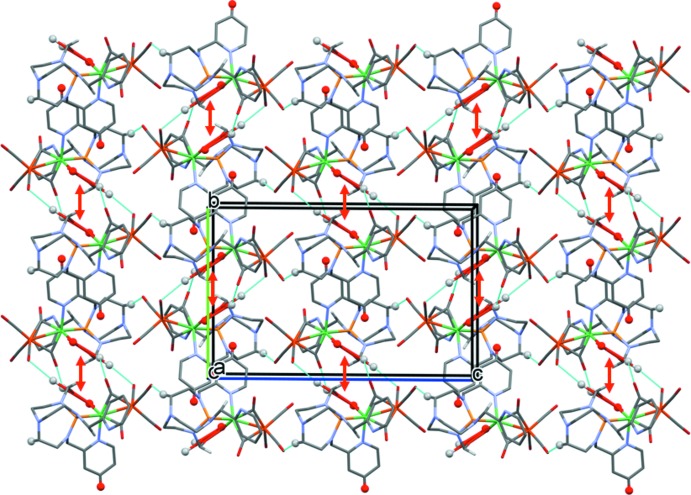
A view along the *a* axis of the crystal packing of complex NiFe(TPAP)(CO)_5_. The hydrogen bonds are shown as cyan dashed lines and the π–π inter­actions as red arrows (Table 2 ▸). Only the H atoms (grey and red balls) involved in these inter­actions have been included. The pyridine rings involved in offset π–π inter­actions are shown in red.

**Figure 3:**
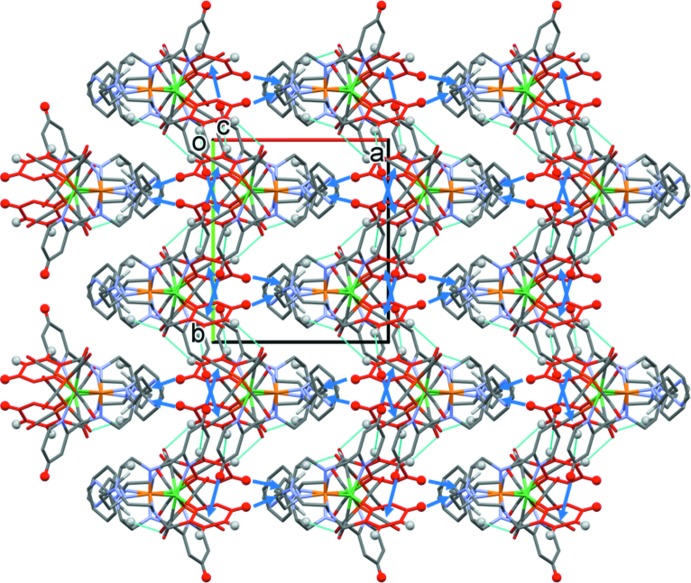
A view along the *c* axis of the crystal packing of complex NiFe(TPAP)(CO)_5_. The hydrogen bonds are shown as cyan dashed lines and the C—H⋯π inter­actions as blue arrows (Table 2 ▸). Only the H atoms (grey and red balls) involved in these inter­actions have been included. The pyridine rings involved in offset π—π inter­actions are shown in red.

In the crystal, complex mol­ecules are linked by C—H⋯O hydrogen bonds and C—H⋯π inter­actions, forming undulating layers parallel to the *bc* plane (Table 2 ▸ and Fig. 2 ▸). Within the layers there are offset π–π inter­actions present involving inversion-related N6/C14–C18 pyridine rings (centroid *Cg*7): *Cg*7⋯*Cg*7^ii^ = 3.6631 (9) Å, inter­planar distance = 3.2739 (5) Å, offset = 1.643 Å, symmetry code: (ii) −*x* + 2, −*y* + 2, −*z* + 1 (Fig. 3 ▸). The layers are linked by further C—H⋯π inter­actions, forming a supra­molecular framework (Table 2 ▸ and Fig. 3 ▸).

## Database survey   

A search was performed to compare previously published structures of mol­ecular NiFe bimetallic complexes that are potential biological mimics of NiFe hydrogenase. Specifically, the search was for mol­ecular NiFe that contained three terminally bound CO or CN ligands to Fe and any bridging ligand(s) between the Ni and Fe metal ions. This search was limited to these features because of their importance in the active site of NiFe hydrogenase. A search of the Cambridge Structural Database (CSD, Version 5.40, update November 2018; Groom *et al.*, 2016 ▸), gave 32 hits with these attributes. Only 12 structures have Ni—Fe bond lengths relatively close (within 0.2 Å) to those of the reduced form of NiFe hydrogenase (∼2.5 Å). However, the NiFe complexes of these 12 structures [CSD refcodes: FANHEK, FANHEK01, FANGUZ, FANHAG, FANHIO and FANHUA (Song *et al.*, 2017 ▸), LAZWEP (Zhu *et al.*, 2005 ▸), SUQQOL (Barton *et al.*, 2009 ▸), UCUXOH and UCUXUN (Carroll *et al.*, 2011 ▸), UQAJAZ (Manor & Rauchfuss, 2013 ▸) and YOKWIE (Walther *et al.*, 1995 ▸); see Table S1 in the supporting information] do not feature a pendant base, which has been demonstrated by Armstrong and collaborators to play a key role in the function of NiFe hydrogenase (Evans *et al.*, 2016 ▸). Structural models of NiFe hydrogenase that incorporate a pendant base but lack the three terminally bound CO or CN ligands of the NiFe hydrogenase active site can be found here [CSD refcodes: EJUSEJ and EJUSUZ (Sun *et al.*, 2016 ▸), FOTKOP (Tanino *et al.*, 2009 ▸) and QEKLAT (Liaw *et al.*, 2000 ▸); see Table S2 in the supporting information].

## Synthesis and crystallization   

The synthesis of NiFe(TPAP)(CO)_5_ is summarized in the reaction scheme. As it is air- and moisture-sensitive, all solvents (except for C_6_D_6_) were first purged with argon and dried using a solvent purification system. Iron^0^ penta­carbonyl was purchased from Sigma–Aldrich and used without further purification. Ni(TPAP)(COD) was synthesized according to an established procedure (Thammavongsy *et al.*, 2018 ▸). ^1^H and ^31^P NMR spectra were recorded on a Bruker AVANCE 600 MHz and were referenced to the residual protio solvent peak (except for ^31^P, which was referenced to the absolute frequency of 0 ppm in the ^1^H dimension according to the Xi scale). Infrared (IR) absorption of the solid NiFe(TPAP)(CO)_5_ was taken on a Thermo Scientific Nicolet iS5 spectrophotometer with an iD5 ATR attachment. Elemental analyses were performed on a PerkinElmer 2400 Series II CHNS elemental analyzer.

**Figure 4:**
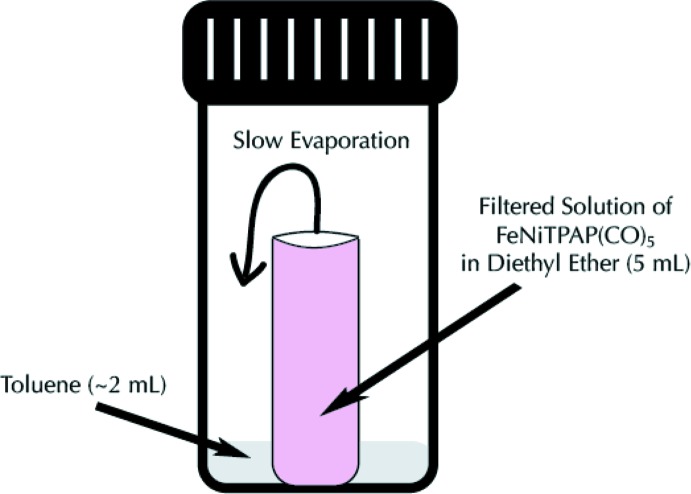
Method of crystallization for NiFe(TPAP)(CO)_5_.

In a glove box, a solution of TPAP (61.2 mg, 0.136 mmol) in 3 ml of tetra­hydro­furan was added to a solution of bis­(1,5-cyclo­octa­diene)nickel(0) (37.4 mg, 0.136 mmol) in tetra­hydro­furan. The solution immediately turned dark forest green and was stirred for 1 h at room temperature. To this solution, iron(0) penta­carbonyl (26.6 mg, 0.136 mmol) in 3 ml of tetra­hydro­furan was added. The solution turned dark orange–brown and was stirred for 1 h. The solvent was removed under vacuum and re-dissolved in diethyl ether. The re-dissolved product was filtered through a glass disposable Pasteur pipette packed with a 25 mm glass microfiber filter and celite (3 cm). The method of crystallization is illustrated in Fig. 4 ▸ and lead to the formation of pink block-like crystals of the title complex (52% yield).

The compound is diamagnetic and was characterized by ^1^H NMR (C_6_D_6_, 600 MHz): 2.45–2.58 (*m*, 12H, NCH_2_CH_2_N), 4.09 (*s*, 6H, PyrCH_2_), 6.58 (*t*, 3H, Pyr), 6.96 (*t*, 3H, Pyr), 7.09 (*t*, 3H, Pyr), 8.93 (*m*, 3H, Pyr). ^31^P{^1^H} NMR (C_6_D_6_, 242.94 MHz): 118.6. IR (C=O): 1745, 1770, 1919 and 2001 cm^−1^. Elemental Analysis for C_29_H_30_FeN_7_NiO_5_P: C, 49.61; H, 4.31; N, 13.96; found: C, 49.52; H, 4.28; N, 13.63.

## Refinement   

Crystal data, data collection and structure refinement details are summarized in Table 3 ▸. The hydrogen atoms were fixed geometrically and allowed to ride on their parent atoms: C—H = 0.95–0.99 Å with *U*_iso_(H) = 1.2*U*_eq_(C).

## Supplementary Material

Crystal structure: contains datablock(s) global, I. DOI: 10.1107/S2056989019003256/su5488sup1.cif

Structure factors: contains datablock(s) I. DOI: 10.1107/S2056989019003256/su5488Isup2.hkl

CSD searches. DOI: 10.1107/S2056989019003256/su5488sup3.pdf

CCDC reference: 1901532

Additional supporting information:  crystallographic information; 3D view; checkCIF report

## Figures and Tables

**Table 1:** Selected geometric parameters (Å, °)

Ni1—Fe1	2.4828 (4)	Ni1—N6	2.1167 (11)
Ni1—C25	1.8983 (13)	Ni1—N7	2.1394 (11)
Ni1—C26	1.9805 (13)	Ni1—P1	2.2276 (4)
			
C28—Fe1—C25	168.88 (6)	C25—Ni1—N7	159.65 (5)
C27—Fe1—C26	144.68 (6)	Ni1—C25—Fe1	80.85 (5)
C26—Ni1—N6	155.94 (5)	Fe1—C26—Ni1	79.42 (5)

**Table 2:** Hydrogen-bond geometry (Å, °) *Cg*6 and *Cg*7 are the centroids of pyridine rings N5/C8–C12 and N6/C14–C18, respectively.

*D*—H⋯*A*	*D*—H	H⋯*A*	*D*⋯*A*	*D*—H⋯*A*
C7—H7*A*⋯O2	0.99	2.49	3.3100 (18)	140
C13—H13*A*⋯O1	0.99	2.21	3.1372 (16)	156
C5—H5*A*⋯O5^i^	0.99	2.51	3.4713 (19)	164
C15—H15⋯O3^ii^	0.95	2.52	3.4048 (17)	156
C16—H16⋯O1^ii^	0.95	2.59	3.4499 (18)	151
C17—H17⋯*Cg*6^iii^	0.95	2.83	3.6231 (16)	142
C22—H22⋯*Cg*7^iv^	0.95	2.99	3.8527 (15)	152

Symmetry codes: (i) 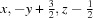; (ii) 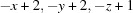; (iii) 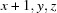; (iv) 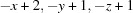.

**Table 3:** Experimental details

Crystal data	
Chemical formula	[FeNi(C_24_H_30_N_7_P)(CO)_5_]
*M*_r_	702.13
Crystal system, space group	Monoclinic, *P*2_1_/*c*
Temperature (K)	88
*a*, *b*, *c* (Å)	11.5584 (15), 12.9709 (17), 20.761 (3)
β (°)	103.1611 (16)
*V* (Å^3^)	3030.8 (7)
*Z*	4
Radiation type	Mo *K*α
μ (mm^−1^)	1.20
Crystal size (mm)	0.35 × 0.34 × 0.23

Data collection	
Diffractometer	Bruker SMART APEXII CCD
Absorption correction	Multi-scan (*SADABS*; Bruker, 2014 ▸)
*T*_min_, *T*_max_	0.654, 0.746
No. of measured, independent and observed [*I* > 2σ(*I*)] reflections	34054, 7655, 6933
*R*_int_	0.026
(sin θ/λ)_max_ (Å^−1^)	0.684

Refinement	
*R*[*F* ^2^ > 2σ(*F* ^2^)], *wR*(*F* ^2^), *S*	0.025, 0.065, 1.04
No. of reflections	7655
No. of parameters	397
H-atom treatment	H-atom parameters constrained
Δρ_max_, Δρ_min_ (e Å^−3^)	0.44, −0.24

Computer programs: *APEX2* and *SAINT* (Bruker, 2014 ▸), *SHELXT2014* (Sheldrick, 2015*a* ▸), *SHELXL2014* (Sheldrick, 2015*b* ▸), *SHELXTL* (Sheldrick, 2008 ▸), *Mercury* (Macrae *et al.*, 2008 ▸) and *PLATON* (Spek, 2009 ▸).

## supplementary crystallographic information

### Crystal data

**Table:**

[FeNi(C_24_H_30_N_7_P)(CO)_5_]	*F*(000) = 1448
*M**_r_* = 702.13	*D*_x_ = 1.539 Mg m^−^^3^
Monoclinic, *P*2_1_/*c*	Mo *K*α radiation, λ = 0.71073 Å
*a* = 11.5584 (15) Å	Cell parameters from 9822 reflections
*b* = 12.9709 (17) Å	θ = 2.4–28.9°
*c* = 20.761 (3) Å	µ = 1.20 mm^−^^1^
β = 103.1611 (16)°	*T* = 88 K
*V* = 3030.8 (7) Å^3^	Block, pink
*Z* = 4	0.35 × 0.34 × 0.23 mm

### Data collection

**Table:**

Bruker SMART APEX II CCD diffractometer	6933 reflections with *I* > 2σ(*I*)
Radiation source: fine-focus sealed tube	*R*_int_ = 0.026
φ and ω scans	θ_max_ = 29.1°, θ_min_ = 1.8°
Absorption correction: multi-scan (SADABS; Bruker, 2014)	*h* = −15→15
*T*_min_ = 0.654, *T*_max_ = 0.746	*k* = −16→16
34054 measured reflections	*l* = −26→26
7655 independent reflections	

### Refinement

**Table:**

Refinement on *F*^2^	Primary atom site location: dual
Least-squares matrix: full	Secondary atom site location: difference Fourier map
*R*[*F*^2^ > 2σ(*F*^2^)] = 0.025	Hydrogen site location: inferred from neighbouring sites
*wR*(*F*^2^) = 0.065	H-atom parameters constrained
*S* = 1.04	*w* = 1/[σ^2^(*F*_o_^2^) + (0.0306*P*)^2^ + 1.444*P*] where *P* = (*F*_o_^2^ + 2*F*_c_^2^)/3
7655 reflections	(Δ/σ)_max_ = 0.003
397 parameters	Δρ_max_ = 0.44 e Å^−^^3^
0 restraints	Δρ_min_ = −0.24 e Å^−^^3^

### Special details

**Table:**

Geometry. All esds (except the esd in the dihedral angle between two l.s. planes) are estimated using the full covariance matrix. The cell esds are taken into account individually in the estimation of esds in distances, angles and torsion angles; correlations between esds in cell parameters are only used when they are defined by crystal symmetry. An approximate (isotropic) treatment of cell esds is used for estimating esds involving l.s. planes.
Refinement. A pink crystal of approximate dimensions 0.230 x 0.342 x 0.354 mm was mounted in a cryoloop and transferred to a Bruker SMART APEX II diffractometer. The APEX2 program package was used to determine the unit-cell parameters and for data collection (30 sec/frame scan time for a sphere of diffraction data). The raw frame data was processed using SAINT and SADABS to yield the reflection data file. Subsequent calculations were carried out using the SHELXTL program. The diffraction symmetry was 2/m and the systematic absences were consistent with the monoclinic space group P21/c that was later determined to be correct.The structure was solved by dual space methods and refined on F2 by full-matrix least-squares techniques. The analytical scattering factors for neutral atoms were used throughout the analysis. Hydrogen atoms were included using a riding model.Least-squares analysis yielded wR2 = 0.0645 and Goof = 1.039 for 397 variables refined against 7655 data, R1 = 0.0249 for those 6933 data with I > 2sigma(I).

### Fractional atomic coordinates and isotropic or equivalent isotropic displacement parameters (Å^2^)

**Table:**

	*x*	*y*	*z*	*U*_iso_*/*U*_eq_	
Ni1	0.78813 (2)	0.75961 (2)	0.57899 (2)	0.01039 (5)	
Fe1	0.83674 (2)	0.81265 (2)	0.69699 (2)	0.01367 (5)	
P1	0.63890 (3)	0.74995 (2)	0.48985 (2)	0.01053 (7)	
O1	0.71929 (9)	0.96779 (7)	0.59941 (5)	0.0198 (2)	
O2	0.67948 (9)	0.62971 (8)	0.66169 (5)	0.0228 (2)	
O3	1.05737 (9)	0.92707 (9)	0.69826 (5)	0.0265 (2)	
O4	0.96879 (14)	0.67309 (10)	0.79969 (6)	0.0465 (4)	
O5	0.70191 (11)	0.93903 (10)	0.77117 (6)	0.0365 (3)	
N1	0.44398 (10)	0.72704 (9)	0.35041 (6)	0.0179 (2)	
N2	0.49806 (10)	0.76746 (8)	0.49634 (6)	0.0138 (2)	
N3	0.65867 (9)	0.83531 (8)	0.43151 (5)	0.0128 (2)	
N4	0.64069 (9)	0.63299 (8)	0.45488 (5)	0.0127 (2)	
N5	0.33082 (10)	0.68828 (9)	0.61849 (6)	0.0190 (2)	
N6	0.90719 (9)	0.82868 (8)	0.52801 (5)	0.0114 (2)	
N7	0.86169 (10)	0.61084 (8)	0.56854 (5)	0.0132 (2)	
C1	0.35566 (12)	0.76163 (11)	0.38478 (7)	0.0207 (3)	
H1A	0.313191	0.700888	0.396825	0.025*	
H1B	0.296821	0.805098	0.354545	0.025*	
C2	0.40834 (11)	0.82334 (10)	0.44759 (7)	0.0169 (3)	
H2A	0.444677	0.887102	0.434913	0.020*	
H2B	0.343065	0.844000	0.468557	0.020*	
C3	0.51347 (12)	0.80175 (10)	0.32364 (7)	0.0180 (3)	
H3A	0.476938	0.870659	0.324344	0.022*	
H3B	0.510531	0.784077	0.276907	0.022*	
C4	0.64332 (12)	0.80724 (10)	0.36131 (6)	0.0154 (2)	
H4A	0.681047	0.739374	0.358493	0.018*	
H4B	0.685152	0.858596	0.339599	0.018*	
C5	0.47613 (12)	0.61929 (10)	0.35048 (7)	0.0178 (3)	
H5A	0.529927	0.609888	0.320029	0.021*	
H5B	0.403319	0.578709	0.332705	0.021*	
C6	0.53703 (11)	0.57515 (10)	0.41838 (7)	0.0155 (2)	
H6A	0.477702	0.571735	0.446001	0.019*	
H6B	0.562387	0.503673	0.412068	0.019*	
C7	0.45393 (12)	0.69685 (10)	0.53978 (7)	0.0159 (3)	
H7A	0.518926	0.649071	0.559918	0.019*	
H7B	0.389244	0.655052	0.512545	0.019*	
C8	0.40765 (12)	0.74722 (10)	0.59474 (7)	0.0157 (3)	
C9	0.44373 (12)	0.84452 (11)	0.61977 (7)	0.0192 (3)	
H9	0.498761	0.883663	0.602136	0.023*	
C10	0.39748 (13)	0.88323 (12)	0.67119 (7)	0.0231 (3)	
H10	0.420615	0.949320	0.689334	0.028*	
C11	0.31717 (13)	0.82395 (12)	0.69555 (7)	0.0235 (3)	
H11	0.283070	0.848991	0.730052	0.028*	
C12	0.28788 (13)	0.72720 (13)	0.66827 (7)	0.0234 (3)	
H12	0.234358	0.686080	0.685815	0.028*	
C13	0.74239 (11)	0.92003 (9)	0.45473 (6)	0.0124 (2)	
H13A	0.721877	0.952193	0.493917	0.015*	
H13B	0.732539	0.973095	0.419624	0.015*	
C14	0.87150 (11)	0.88702 (9)	0.47288 (6)	0.0114 (2)	
C15	0.95007 (11)	0.91641 (9)	0.43428 (6)	0.0137 (2)	
H15	0.922438	0.955186	0.394968	0.016*	
C16	1.06887 (12)	0.88868 (10)	0.45363 (7)	0.0159 (2)	
H16	1.123360	0.907402	0.427608	0.019*	
C17	1.10652 (11)	0.83317 (10)	0.51164 (7)	0.0153 (2)	
H17	1.187851	0.815395	0.527082	0.018*	
C18	1.02295 (11)	0.80406 (10)	0.54675 (6)	0.0134 (2)	
H18	1.048898	0.764760	0.585996	0.016*	
C19	0.75915 (11)	0.59715 (10)	0.45125 (6)	0.0140 (2)	
H19A	0.804308	0.656203	0.439275	0.017*	
H19B	0.750656	0.545493	0.415367	0.017*	
C20	0.83023 (11)	0.54968 (10)	0.51464 (6)	0.0128 (2)	
C21	0.86477 (11)	0.44644 (10)	0.51565 (7)	0.0157 (2)	
H21	0.839128	0.404559	0.477526	0.019*	
C22	0.93683 (12)	0.40518 (10)	0.57267 (7)	0.0186 (3)	
H22	0.960609	0.334938	0.574257	0.022*	
C23	0.97318 (12)	0.46874 (10)	0.62711 (7)	0.0189 (3)	
H23	1.024363	0.443586	0.666413	0.023*	
C24	0.93330 (12)	0.56995 (10)	0.62305 (7)	0.0172 (3)	
H24	0.957788	0.612864	0.660774	0.021*	
C25	0.75950 (11)	0.88650 (10)	0.61819 (6)	0.0144 (2)	
C26	0.73686 (12)	0.70277 (10)	0.65656 (7)	0.0166 (3)	
C27	0.97118 (12)	0.88218 (10)	0.69752 (6)	0.0168 (3)	
C28	0.91548 (15)	0.72597 (12)	0.75931 (7)	0.0258 (3)	
C29	0.75277 (13)	0.88608 (12)	0.74254 (7)	0.0223 (3)	

### Atomic displacement parameters (Å^2^)

**Table:**

	*U*^11^	*U*^22^	*U*^33^	*U*^12^	*U*^13^	*U*^23^
Ni1	0.01101 (8)	0.00995 (8)	0.01015 (8)	0.00006 (5)	0.00225 (6)	0.00090 (5)
Fe1	0.01742 (10)	0.01384 (9)	0.01018 (9)	−0.00202 (7)	0.00400 (7)	0.00039 (6)
P1	0.00903 (15)	0.00987 (14)	0.01244 (15)	−0.00003 (10)	0.00189 (11)	0.00160 (11)
O1	0.0256 (5)	0.0146 (4)	0.0196 (5)	0.0043 (4)	0.0062 (4)	0.0003 (4)
O2	0.0267 (5)	0.0211 (5)	0.0213 (5)	−0.0078 (4)	0.0066 (4)	0.0037 (4)
O3	0.0194 (5)	0.0317 (6)	0.0281 (6)	−0.0041 (4)	0.0045 (4)	0.0057 (5)
O4	0.0709 (10)	0.0367 (7)	0.0242 (6)	0.0018 (7)	−0.0055 (6)	0.0153 (5)
O5	0.0357 (7)	0.0463 (7)	0.0336 (6)	0.0003 (6)	0.0205 (5)	−0.0122 (6)
N1	0.0166 (6)	0.0152 (5)	0.0202 (6)	−0.0025 (4)	0.0009 (5)	0.0033 (4)
N2	0.0096 (5)	0.0132 (5)	0.0187 (5)	0.0009 (4)	0.0035 (4)	0.0049 (4)
N3	0.0122 (5)	0.0135 (5)	0.0118 (5)	−0.0035 (4)	0.0006 (4)	0.0016 (4)
N4	0.0105 (5)	0.0114 (5)	0.0147 (5)	0.0006 (4)	−0.0001 (4)	−0.0006 (4)
N5	0.0132 (5)	0.0264 (6)	0.0169 (6)	−0.0024 (4)	0.0021 (4)	0.0032 (5)
N6	0.0120 (5)	0.0109 (5)	0.0115 (5)	−0.0010 (4)	0.0027 (4)	−0.0003 (4)
N7	0.0136 (5)	0.0119 (5)	0.0133 (5)	0.0006 (4)	0.0013 (4)	0.0015 (4)
C1	0.0116 (6)	0.0223 (7)	0.0254 (7)	−0.0005 (5)	−0.0016 (5)	0.0061 (5)
C2	0.0112 (6)	0.0166 (6)	0.0227 (7)	0.0034 (5)	0.0032 (5)	0.0051 (5)
C3	0.0179 (7)	0.0174 (6)	0.0153 (6)	−0.0043 (5)	−0.0033 (5)	0.0042 (5)
C4	0.0162 (6)	0.0167 (6)	0.0121 (6)	−0.0026 (5)	0.0007 (5)	0.0024 (5)
C5	0.0174 (6)	0.0163 (6)	0.0167 (6)	−0.0044 (5)	−0.0023 (5)	0.0007 (5)
C6	0.0151 (6)	0.0123 (6)	0.0175 (6)	−0.0035 (5)	0.0000 (5)	0.0002 (5)
C7	0.0129 (6)	0.0138 (6)	0.0216 (7)	−0.0012 (5)	0.0055 (5)	0.0032 (5)
C8	0.0098 (6)	0.0190 (6)	0.0173 (6)	0.0023 (5)	0.0011 (5)	0.0045 (5)
C9	0.0152 (6)	0.0184 (6)	0.0230 (7)	0.0026 (5)	0.0021 (5)	0.0031 (5)
C10	0.0229 (7)	0.0224 (7)	0.0206 (7)	0.0081 (6)	−0.0023 (6)	−0.0001 (5)
C11	0.0176 (7)	0.0382 (8)	0.0130 (6)	0.0097 (6)	0.0002 (5)	0.0006 (6)
C12	0.0138 (6)	0.0393 (8)	0.0163 (7)	−0.0006 (6)	0.0016 (5)	0.0049 (6)
C13	0.0112 (6)	0.0106 (5)	0.0142 (6)	−0.0012 (4)	0.0009 (4)	0.0027 (4)
C14	0.0122 (6)	0.0096 (5)	0.0117 (6)	−0.0024 (4)	0.0014 (4)	−0.0014 (4)
C15	0.0166 (6)	0.0126 (5)	0.0122 (6)	−0.0028 (5)	0.0039 (5)	0.0000 (4)
C16	0.0162 (6)	0.0158 (6)	0.0182 (6)	−0.0042 (5)	0.0090 (5)	−0.0028 (5)
C17	0.0108 (6)	0.0150 (6)	0.0203 (6)	−0.0001 (5)	0.0037 (5)	−0.0028 (5)
C18	0.0129 (6)	0.0130 (6)	0.0139 (6)	0.0007 (4)	0.0019 (5)	−0.0003 (4)
C19	0.0138 (6)	0.0150 (6)	0.0129 (6)	0.0028 (5)	0.0023 (5)	0.0002 (5)
C20	0.0109 (6)	0.0141 (6)	0.0136 (6)	0.0003 (4)	0.0032 (4)	0.0007 (5)
C21	0.0142 (6)	0.0146 (6)	0.0178 (6)	0.0004 (5)	0.0025 (5)	−0.0024 (5)
C22	0.0191 (7)	0.0132 (6)	0.0231 (7)	0.0037 (5)	0.0038 (5)	0.0022 (5)
C23	0.0198 (7)	0.0179 (6)	0.0168 (6)	0.0051 (5)	−0.0003 (5)	0.0047 (5)
C24	0.0197 (7)	0.0161 (6)	0.0138 (6)	0.0019 (5)	−0.0004 (5)	0.0003 (5)
C25	0.0144 (6)	0.0160 (6)	0.0142 (6)	−0.0019 (5)	0.0059 (5)	−0.0005 (5)
C26	0.0167 (6)	0.0179 (6)	0.0155 (6)	−0.0002 (5)	0.0044 (5)	0.0013 (5)
C27	0.0202 (7)	0.0180 (6)	0.0119 (6)	0.0037 (5)	0.0027 (5)	0.0028 (5)
C28	0.0376 (9)	0.0222 (7)	0.0160 (7)	−0.0068 (6)	0.0026 (6)	0.0020 (6)
C29	0.0233 (7)	0.0274 (7)	0.0177 (7)	−0.0060 (6)	0.0079 (6)	−0.0021 (6)

### Geometric parameters (Å, º)

**Table:**

Ni1—Fe1	2.4828 (4)	C3—H3B	0.9900
Ni1—C25	1.8983 (13)	C4—H4A	0.9900
Ni1—C26	1.9805 (13)	C4—H4B	0.9900
Ni1—N6	2.1167 (11)	C5—C6	1.5358 (18)
Ni1—N7	2.1394 (11)	C5—H5A	0.9900
Ni1—P1	2.2276 (4)	C5—H5B	0.9900
Fe1—C29	1.7781 (15)	C6—H6A	0.9900
Fe1—C27	1.7946 (14)	C6—H6B	0.9900
Fe1—C28	1.7971 (16)	C7—C8	1.5143 (19)
Fe1—C26	1.9046 (14)	C7—H7A	0.9900
Fe1—C25	1.9304 (13)	C7—H7B	0.9900
P1—N2	1.6792 (12)	C8—C9	1.3921 (19)
P1—N4	1.6841 (11)	C9—C10	1.392 (2)
P1—N3	1.6944 (11)	C9—H9	0.9500
P1—N1	3.2518 (13)	C10—C11	1.386 (2)
O1—C25	1.1821 (16)	C10—H10	0.9500
O2—C26	1.1754 (17)	C11—C12	1.387 (2)
O3—C27	1.1509 (17)	C11—H11	0.9500
O4—C28	1.148 (2)	C12—H12	0.9500
O5—C29	1.1531 (19)	C13—C14	1.5153 (17)
N1—C1	1.4438 (19)	C13—H13A	0.9900
N1—C5	1.4461 (17)	C13—H13B	0.9900
N1—C3	1.4479 (17)	C14—C15	1.3941 (17)
N2—C7	1.4566 (16)	C15—C16	1.3875 (19)
N2—C2	1.4653 (17)	C15—H15	0.9500
N3—C13	1.4711 (16)	C16—C17	1.3855 (19)
N3—C4	1.4731 (16)	C16—H16	0.9500
N4—C19	1.4640 (16)	C17—C18	1.3882 (18)
N4—C6	1.4693 (16)	C17—H17	0.9500
N5—C12	1.343 (2)	C18—H18	0.9500
N5—C8	1.3474 (17)	C19—C20	1.5146 (17)
N6—C18	1.3441 (16)	C19—H19A	0.9900
N6—C14	1.3556 (16)	C19—H19B	0.9900
N7—C24	1.3492 (17)	C20—C21	1.3962 (17)
N7—C20	1.3522 (16)	C21—C22	1.3902 (19)
C1—C2	1.533 (2)	C21—H21	0.9500
C1—H1A	0.9900	C22—C23	1.385 (2)
C1—H1B	0.9900	C22—H22	0.9500
C2—H2A	0.9900	C23—C24	1.3875 (18)
C2—H2B	0.9900	C23—H23	0.9500
C3—C4	1.5280 (18)	C24—H24	0.9500
C3—H3A	0.9900		
			
C28—Fe1—C25	168.88 (6)	N3—C4—H4B	108.8
C27—Fe1—C26	144.68 (6)	C3—C4—H4B	108.8
C26—Ni1—N6	155.94 (5)	H4A—C4—H4B	107.7
C25—Ni1—N7	159.65 (5)	N1—C5—C6	115.16 (11)
Ni1—C25—Fe1	80.85 (5)	N1—C5—H5A	108.5
Fe1—C26—Ni1	79.42 (5)	C6—C5—H5A	108.5
C25—Ni1—C26	81.98 (6)	N1—C5—H5B	108.5
C25—Ni1—N6	92.52 (5)	C6—C5—H5B	108.5
C26—Ni1—N7	86.91 (5)	H5A—C5—H5B	107.5
N6—Ni1—N7	90.71 (4)	N4—C6—C5	115.63 (10)
C25—Ni1—P1	103.14 (4)	N4—C6—H6A	108.4
C26—Ni1—P1	109.64 (4)	C5—C6—H6A	108.4
N6—Ni1—P1	94.41 (3)	N4—C6—H6B	108.4
N7—Ni1—P1	96.63 (3)	C5—C6—H6B	108.4
C25—Ni1—Fe1	50.14 (4)	H6A—C6—H6B	107.4
C26—Ni1—Fe1	48.94 (4)	N2—C7—C8	115.39 (11)
N6—Ni1—Fe1	110.45 (3)	N2—C7—H7A	108.4
N7—Ni1—Fe1	110.13 (3)	C8—C7—H7A	108.4
P1—Ni1—Fe1	142.544 (13)	N2—C7—H7B	108.4
C29—Fe1—C27	107.71 (6)	C8—C7—H7B	108.4
C29—Fe1—C28	101.57 (7)	H7A—C7—H7B	107.5
C27—Fe1—C28	90.81 (7)	N5—C8—C9	122.97 (13)
C29—Fe1—C26	106.61 (6)	N5—C8—C7	113.97 (12)
C28—Fe1—C26	90.34 (7)	C9—C8—C7	123.04 (12)
C29—Fe1—C25	88.96 (6)	C10—C9—C8	118.59 (13)
C27—Fe1—C25	89.34 (6)	C10—C9—H9	120.7
C26—Fe1—C25	83.15 (6)	C8—C9—H9	120.7
C29—Fe1—Ni1	130.16 (5)	C11—C10—C9	119.06 (14)
C27—Fe1—Ni1	98.42 (4)	C11—C10—H10	120.5
C28—Fe1—Ni1	120.04 (5)	C9—C10—H10	120.5
C26—Fe1—Ni1	51.64 (4)	C10—C11—C12	118.31 (14)
C25—Fe1—Ni1	49.01 (4)	C10—C11—H11	120.8
N2—P1—N4	105.29 (6)	C12—C11—H11	120.8
N2—P1—N3	105.07 (5)	N5—C12—C11	123.78 (14)
N4—P1—N3	105.28 (5)	N5—C12—H12	118.1
N2—P1—Ni1	120.47 (4)	C11—C12—H12	118.1
N4—P1—Ni1	109.00 (4)	N3—C13—C14	114.05 (10)
N3—P1—Ni1	110.62 (4)	N3—C13—H13A	108.7
N2—P1—N1	66.17 (5)	C14—C13—H13A	108.7
N4—P1—N1	66.91 (4)	N3—C13—H13B	108.7
N3—P1—N1	66.56 (4)	C14—C13—H13B	108.7
Ni1—P1—N1	173.31 (3)	H13A—C13—H13B	107.6
C1—N1—C5	120.69 (11)	N6—C14—C15	121.67 (11)
C1—N1—C3	119.87 (11)	N6—C14—C13	117.44 (10)
C5—N1—C3	118.84 (12)	C15—C14—C13	120.88 (11)
C1—N1—P1	87.48 (8)	C16—C15—C14	119.60 (12)
C5—N1—P1	87.80 (7)	C16—C15—H15	120.2
C3—N1—P1	87.03 (7)	C14—C15—H15	120.2
C7—N2—C2	116.50 (10)	C17—C16—C15	118.73 (12)
C7—N2—P1	116.56 (9)	C17—C16—H16	120.6
C2—N2—P1	123.92 (9)	C15—C16—H16	120.6
C13—N3—C4	115.52 (10)	C16—C17—C18	118.65 (12)
C13—N3—P1	116.08 (8)	C16—C17—H17	120.7
C4—N3—P1	122.69 (8)	C18—C17—H17	120.7
C19—N4—C6	118.27 (10)	N6—C18—C17	123.31 (12)
C19—N4—P1	114.31 (8)	N6—C18—H18	118.3
C6—N4—P1	126.42 (9)	C17—C18—H18	118.3
C12—N5—C8	117.27 (13)	N4—C19—C20	114.39 (10)
C18—N6—C14	117.94 (11)	N4—C19—H19A	108.7
C18—N6—Ni1	118.19 (8)	C20—C19—H19A	108.7
C14—N6—Ni1	123.43 (8)	N4—C19—H19B	108.7
C24—N7—C20	117.48 (11)	C20—C19—H19B	108.7
C24—N7—Ni1	116.85 (9)	H19A—C19—H19B	107.6
C20—N7—Ni1	125.09 (8)	N7—C20—C21	121.89 (12)
N1—C1—C2	113.21 (11)	N7—C20—C19	118.11 (11)
N1—C1—H1A	108.9	C21—C20—C19	119.97 (11)
C2—C1—H1A	108.9	C22—C21—C20	119.72 (12)
N1—C1—H1B	108.9	C22—C21—H21	120.1
C2—C1—H1B	108.9	C20—C21—H21	120.1
H1A—C1—H1B	107.7	C23—C22—C21	118.52 (12)
N2—C2—C1	114.26 (11)	C23—C22—H22	120.7
N2—C2—H2A	108.7	C21—C22—H22	120.7
C1—C2—H2A	108.7	C22—C23—C24	118.61 (12)
N2—C2—H2B	108.7	C22—C23—H23	120.7
C1—C2—H2B	108.7	C24—C23—H23	120.7
H2A—C2—H2B	107.6	N7—C24—C23	123.70 (12)
N1—C3—C4	113.62 (11)	N7—C24—H24	118.2
N1—C3—H3A	108.8	C23—C24—H24	118.2
C4—C3—H3A	108.8	O1—C25—Ni1	136.54 (11)
N1—C3—H3B	108.8	O1—C25—Fe1	142.51 (11)
C4—C3—H3B	108.8	O2—C26—Fe1	149.46 (11)
H3A—C3—H3B	107.7	O2—C26—Ni1	130.79 (11)
N3—C4—C3	113.63 (11)	O3—C27—Fe1	179.54 (13)
N3—C4—H4A	108.8	O4—C28—Fe1	177.65 (15)
C3—C4—H4A	108.8	O5—C29—Fe1	175.78 (14)
			
N4—P1—N2—C7	64.08 (11)	N2—C7—C8—C9	−24.33 (18)
N3—P1—N2—C7	174.98 (9)	N5—C8—C9—C10	−0.7 (2)
Ni1—P1—N2—C7	−59.46 (11)	C7—C8—C9—C10	−178.84 (13)
N1—P1—N2—C7	119.67 (10)	C8—C9—C10—C11	−0.1 (2)
N4—P1—N2—C2	−95.51 (11)	C9—C10—C11—C12	1.2 (2)
N3—P1—N2—C2	15.39 (12)	C8—N5—C12—C11	0.7 (2)
Ni1—P1—N2—C2	140.95 (9)	C10—C11—C12—N5	−1.5 (2)
N1—P1—N2—C2	−39.92 (10)	C4—N3—C13—C14	−82.37 (13)
N2—P1—N3—C13	112.78 (9)	P1—N3—C13—C14	71.80 (12)
N4—P1—N3—C13	−136.32 (9)	C18—N6—C14—C15	3.48 (17)
Ni1—P1—N3—C13	−18.71 (10)	Ni1—N6—C14—C15	−168.78 (9)
N1—P1—N3—C13	167.84 (10)	C18—N6—C14—C13	−176.16 (11)
N2—P1—N3—C4	−95.07 (11)	Ni1—N6—C14—C13	11.58 (15)
N4—P1—N3—C4	15.83 (11)	N3—C13—C14—N6	−70.00 (14)
Ni1—P1—N3—C4	133.44 (9)	N3—C13—C14—C15	110.36 (13)
N1—P1—N3—C4	−40.01 (9)	N6—C14—C15—C16	−2.31 (19)
N2—P1—N4—C19	−173.60 (9)	C13—C14—C15—C16	177.31 (11)
N3—P1—N4—C19	75.66 (10)	C14—C15—C16—C17	−0.75 (19)
Ni1—P1—N4—C19	−43.04 (9)	C15—C16—C17—C18	2.47 (19)
N1—P1—N4—C19	131.28 (9)	C14—N6—C18—C17	−1.67 (18)
N2—P1—N4—C6	18.13 (12)	Ni1—N6—C18—C17	171.00 (10)
N3—P1—N4—C6	−92.61 (11)	C16—C17—C18—N6	−1.3 (2)
Ni1—P1—N4—C6	148.69 (10)	C6—N4—C19—C20	−109.08 (13)
N1—P1—N4—C6	−36.99 (10)	P1—N4—C19—C20	81.62 (12)
C5—N1—C1—C2	108.47 (14)	C24—N7—C20—C21	3.25 (18)
C3—N1—C1—C2	−62.59 (16)	Ni1—N7—C20—C21	−167.68 (9)
P1—N1—C1—C2	22.53 (10)	C24—N7—C20—C19	−174.58 (11)
C7—N2—C2—C1	−82.09 (14)	Ni1—N7—C20—C19	14.49 (16)
P1—N2—C2—C1	77.51 (14)	N4—C19—C20—N7	−64.57 (15)
N1—C1—C2—N2	−58.08 (15)	N4—C19—C20—C21	117.56 (13)
C1—N1—C3—C4	108.77 (14)	N7—C20—C21—C22	−2.2 (2)
C5—N1—C3—C4	−62.46 (16)	C19—C20—C21—C22	175.61 (12)
P1—N1—C3—C4	23.39 (11)	C20—C21—C22—C23	−0.5 (2)
C13—N3—C4—C3	−129.51 (11)	C21—C22—C23—C24	1.9 (2)
P1—N3—C4—C3	78.20 (13)	C20—N7—C24—C23	−1.8 (2)
N1—C3—C4—N3	−59.78 (15)	Ni1—N7—C24—C23	169.90 (11)
C1—N1—C5—C6	−65.16 (16)	C22—C23—C24—N7	−0.8 (2)
C3—N1—C5—C6	105.99 (14)	C26—Ni1—C25—O1	−141.55 (16)
P1—N1—C5—C6	20.60 (11)	N6—Ni1—C25—O1	61.98 (15)
C19—N4—C6—C5	−97.58 (13)	N7—Ni1—C25—O1	160.87 (12)
P1—N4—C6—C5	70.27 (15)	P1—Ni1—C25—O1	−33.15 (15)
N1—C5—C6—N4	−52.43 (16)	Fe1—Ni1—C25—O1	176.74 (18)
C2—N2—C7—C8	−75.67 (15)	C26—Ni1—C25—Fe1	41.71 (5)
P1—N2—C7—C8	123.20 (11)	N6—Ni1—C25—Fe1	−114.76 (4)
C12—N5—C8—C9	0.4 (2)	N7—Ni1—C25—Fe1	−15.87 (16)
C12—N5—C8—C7	178.71 (12)	P1—Ni1—C25—Fe1	150.12 (3)
N2—C7—C8—N5	157.37 (11)		

### Hydrogen-bond geometry (Å, º)

*Cg*6 and *Cg*7 are the centroids of pyridine rings N5/C8–C12 and N6/C14–C18, respectively.

**Table:**

*D*—H···*A*	*D*—H	H···*A*	*D*···*A*	*D*—H···*A*
C7—H7*A*···O2	0.99	2.49	3.3100 (18)	140
C13—H13*A*···O1	0.99	2.21	3.1372 (16)	156
C5—H5*A*···O5^i^	0.99	2.51	3.4713 (19)	164
C15—H15···O3^ii^	0.95	2.52	3.4048 (17)	156
C16—H16···O1^ii^	0.95	2.59	3.4499 (18)	151
C17—H17···*Cg*6^iii^	0.95	2.83	3.6231 (16)	142
C22—H22···*Cg*7^iv^	0.95	2.99	3.8527 (15)	152

Symmetry codes: (i) *x*, −*y*+3/2, *z*−1/2; (ii) −*x*+2, −*y*+2, −*z*+1; (iii) *x*+1, *y*, *z*; (iv) −*x*+2, −*y*+1, −*z*+1.
