# Supplementary material for: Crystal structure of NiFe(CO)5[tris(pyridyl­meth­yl)aza­phosphatrane]: a synthetic mimic of the NiFe hydrogenase active site incorporating a pendant pyridine base
Source: Acta Crystallogr E Crystallogr Commun. 2019 Mar 11;75(Pt 4):438–42. doi: 10.1107/S2056989019003256 (PMC6509684; doi:10.1107/S2056989019003256)
Supplement: Supplementary file 3 [file e-75-00438-sup3.pdf]

Crystal structure of $NiFe(CO)_5$(tri(pyridylmethyl)azaphosphatrane): a synthetic mimic of the NiFe hydrogenase active site incorporating a pendant pyridine base

Natwara Sutthirat, Joseph W. Ziller, Jenny Y. Yang and Zachary Thammavongsy*

Table S1. List of NiFe complexes with Ni-Fe bond distances.

**CSD refcodes**	**Ni-Fe distance (Å)**	**Ref**
FANHEK	2.621	1 (Song *et al.*, 2017)
FANHEK01	2.614	1 (Song *et al.*, 2017)
FANGUZ	2.460	1 (Song *et al.*, 2017)
FANHIO	2.650	1 (Song *et al.*, 2017)
FANHUA	2.660	1 (Song *et al.*, 2017)
LAZWEP	2.467	2 (Zhu *et al.*, 2005)
SUQQOL	2.613	3 (Barton *et al.*, 2009)
UCUXOH	2.684	4 (Carroll *et al.*, 2011)
UCUXUN	2.596	4 (Carroll *et al.*, 2011)
UQAJAZ	2.550	5 (Manor & Rauchfuss, 2013)
YOKWIE	2.447	6 (Walther *et al.*, 1995)

Table S2. List of NiFe complexes with Ni-Fe bond distances.

CSD refcodes	Ni-Fe distance (Å)	Ref
EJUSEJ	2.572	7 (Sun *et al.*, 2016)
EJUSUZ	3.164*	7 (Sun *et al.*, 2016)
FOTKOP	3.296	8 (Tanino *et al.*, 2009)
QEKLAT	2.800	9 (Liaw *et al.*, 2000)
FANHUA	2.660	1 (Song *et al.*, 2017)

- Average of two structures in the unit cell.

Reference:

1 Song, L.-C., Lu, Y., Zhu, L. & Li, Q.-L. (2017). *Organometallics* **36**, 750-760.

2 Zhu, W., Marr, A. C., Wang, Q., Neese, F., Spencer, D. J. E., Blake, A. J., Cooke, P. A., Wilson, C. & Schröder, M. (2005). **102**, 18280-18285.

3 Barton, B. E., Whaley, C. M., Rauchfuss, T. B. & Gray, D. L. (2009). *Journal of the American Chemical Society* **131**, 6942-6943.

4 Carroll, M. E., Barton, B. E., Gray, D. L., Mack, A. E. & Rauchfuss, T. B. (2011). *Inorganic Chemistry* **50**, 9554-9563.

5 Manor, B. C. & Rauchfuss, T. B. (2013). *Journal of the American Chemical Society* **135**, 11895-11900.

6 Walther, D., Geßler, S. & Sieler, J. (1995). Zeitschrift für anorganische und allgemeine Chemie **621**, 635-639.

7 Sun, P., Yang, D., Li, Y., Zhang, Y., Su, L., Wang, B. & Qu, J. (2016). *Organometallics* **35**, 751-757.

8 Tanino, S., Li, Z., Ohki, Y. & Tatsumi, K. (2009). *Inorganic Chemistry* **48**, 2358-2360.

9 Liaw, W.-F., Chiang, C.-Y., Lee, G.-H., Peng, S.-M., Lai, C.-H. & Darensbourg, M. Y. (2000). *Inorganic Chemistry* **39**, 480-484.
